# Inhalation of nicotine-containing electronic cigarette vapor exacerbates the features of COPD by inducing ferroptosis in βENaC-overexpressing mice

**DOI:** 10.3389/fimmu.2024.1429946

**Published:** 2024-06-14

**Authors:** Hongwei Han, Maureen Meister, Guangda Peng, Yi Yuan, Jingjuan Qiao, Jenny J. Yang, Zhi-Ren Liu, Xiangming Ji

**Affiliations:** ^1^ Department of Biology, Georgia State University, Atlanta, GA, United States; ^2^ Department of Nutrition, Georgia State University, Atlanta, GA, United States; ^3^ Department of Chemistry, Georgia State University, Atlanta, GA, United States

**Keywords:** fibrosis, ENDS (electronic nicotine delivery systems), COPD (chronic obstructive pulmonary disease), ferroptosis, lipid dysregulations

## Abstract

**Introduction:**

Chronic obstructive pulmonary disease (COPD) is currently listed as the 3^rd^ leading cause of death in the United States. Accumulating data shows the association between COPD occurrence and the usage of electronic nicotine delivery systems (ENDS) in patients. However, the underlying pathogenesis mechanisms of COPD have not been fully understood.

**Methods:**

In the current study, bENaC-overexpressing mice (bENaC mice) were subjected to whole-body ENDS exposure. COPD related features including emphysema, mucus accumulation, inflammation and fibrosis are examined by tissue staining, FACS analysis, cytokine measurement. Cell death and ferroptosis of alveolar epithelial cells were further evaluated by multiple assays including staining, FACS analysis and lipidomics.

**Results:**

ENDS-exposed mice displayed enhanced emphysema and mucus accumulation, suggesting that ENDS exposure promotes COPD features. ENDS exposure also increased immune cell number infiltration in bronchoalveolar lavage and levels of multiple COPD-related cytokines in the lungs, including CCL2, IL-4, IL-13, IL-10, M-CSF, and TNF-α. Moreover, we observed increased fibrosis in ENDS-exposed mice, as evidenced by elevated collagen deposition and a-SMA+ myofibroblast accumulation. By investigating possible mechanisms for how ENDS promoted COPD, we demonstrated that ENDS exposure induced cell death of alveolar epithelial cells, evidenced by TUNEL staining and Annexin V/PI FACS analysis. Furthermore, we identified that ENDS exposure caused lipid dysregulations, including TAGs (9 species) and phospholipids (34 species). As most of these lipid species are highly associated with ferroptosis, we confirmed ENDS also enhanced ferroptosis marker CD71 in both type I and type II alveolar epithelial cells.

**Discussion:**

Overall, our data revealed that ENDS exposure exacerbates features of COPD in bENaC mice including emphysema, mucus accumulation, abnormal lung inflammation, and fibrosis, which involves the effect of COPD development by inducing ferroptosis in the lung.

## Introduction

Chronic obstructive pulmonary disease (COPD) is a primary leading cause of death with limited treatment options, accounting for more than 3 million deaths worldwide annually (1). The phenotypes of COPD are characterized by limitations in expiratory airflow, emphysematous destruction of the lungs, chronic bronchitis, and inflammation of the lung tissue. Patients with COPD experience a variety of symptoms including shortness of breath, wheezing, and/or a chronic cough (2). Cigarette smoking is the major risk factor for COPD. While cigarette smoking in the U.S. has decreased over the past 50 years, there has been a dramatic increase in the usage of electronic nicotine delivery systems (ENDS) as substitutes (1). As ENDS are perceived as safer than traditional cigarettes, they are often used to assist in smoking cessation. The risk of cigarette smoking and its contribution to chronic lung diseases has been well characterized. However, the impact of ENDS on COPD development remains poorly understood (3). Therefore, a thorough understanding of the effects of ENDS usage on chronic lung conditions is essential.

ENDS include vape pens, hookah pens, and electronic cigarettes (e-cigarettes or e-cigs), which transport noncombustible vaporized nicotine products to the lungs (4). The common components of e-cigarette liquid include nicotine, glycerin, propylene glycol, flavorings, and other ingredients. When heated, the nicotine-containing e-cigarette liquid generates vapor, which will be inhaled into the lungs. Vaporization of e-liquid generates toxic compounds similar to cigarette smoke. (Rahman) Reactive aldehydes, such as formaldehyde and acrolein, are common products of the thermal decomposition of propylene glycol and glycerol, the major vehicle components of e-liquids (5). Notably, reactive aldehydes generated from ENDS have been noted in much greater concentrations than recommended occupational safety standards (6). Accumulating data suggest that e-cigarette vapor induces oxidative stress and inflammation *in vitro* (7, 8) and *in vivo* (9, 10). For example, e-cigarette liquid alone caused morphology changes in lung epithelial cells, by initiating a stress phenotype and inflammatory response (9). As a major component of ENDS, nicotine, the addictive component in cigarettes and e-cigarettes, inhibits mucus hydration (11) and induces pro-inflammatory dendritic cell responses (12). Similar results were evidenced in mice where e-cigarette vapor induced airway responses similar to cigarette smoke, including airway inflammation, tissue remodeling, and enhanced mucin expression following four months of exposure (10). These findings suggest the ability of nicotine-containing e-cigarettes could induce physiological changes akin to human COPD.

As the primary risk factor for COPD, cigarette smoking is widely used to study the pathogenesis of COPD in various animal models (13). Despite the recent popularity of ENDS, there are limited investigations aimed at elucidating the effects of e-cigarettes on the development and progression of COPD (4). Therefore, there is an unmet need to study the effects of ENDS, e-cigarettes in particular, on COPD progression utilizing the proper animal model with pre-existing phenotypes of COPD. In this study, we utilized the βENaC mouse, a transgenic line that exhibits defective airway mucus clearance due to the overexpression of the epithelial sodium channel non-voltage gated 1, beta subunit (*Scnn1b)* (14–16). βENaC mice have been used in the investigation of respiratory diseases including COPD and cystic fibrosis (17, 18). Because this strain of mice has symptoms of emphysema and mucus obstruction (17), we postulated that ENDS could exacerbate the phenotypic response of COPD following acute e-cigarette vapor exposure. This study was to evaluate the effects of nicotine-containing e-cigarette vapor in the development and progression of a COPD phenotype including 1) inflammatory response in the lung tissues, 2) mucus accumulation within the bronchioles, 3) small airway fibrosis, and 4) destruction and degradation of alveolar structure; and also investigate the potential mechanisms underneath.

## Materials and methods

### Animals and genotyping

Male and female βENaC were purchased from The Jackson Laboratory (congenic C57BL/6J background, Stock No. 006438, Bar Harbor, ME). Mice were housed in a specific pathogen-free facility on a 12-hour light/dark cycle with free access to water and a standard chow diet throughout treatment. All animal procedures described were approved by the Institutional Animal Care and Use Committee (IACUC) at Georgia State University. To have enough animals for this study, βENaC mice were bred with wild-type C57BL/6J mice to produce an appropriate number of hemizygous βEnaC for our studies. Before weaning age, animals were genotyped to determine the presence of *Scnn1b* overexpression. Briefly, tail snips were collected and lysed in Proteinase K overnight at 56°C in preparation for DNA extraction. DNA was extracted by Dneasy Blood and Tissue kit (Qiagen, Foster City, CA) in preparation for polymerase chain reaction (PCR). PCR was performed using primers specific for *Scnn1b* gene expression as suggested (Forward: CCTCCAAGAGTTCAACTACCG; Reverse: TCTACCAGCTCAGCCACAGTG) (16). To verify the expression of *the Scnn1b* gene, samples were run on a 4% agarose gel containing ethidium bromide. Mice identified as possessing *Scnn1b* overexpression were tagged as βENaC mice for future studies.

### ENDS exposure to βENaC mice

**Figure 1:**
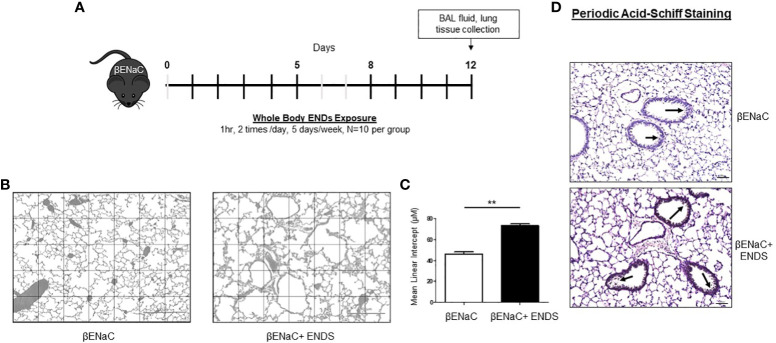
ENDS exposure promoted emphysema and mucus accumulation in βENaC mice. **(A)** Experimental schematic of whole-body ENDS exposure in βENaC mice. **(B)** ENDS exposure caused emphysematous phenotype changes in βENaC mice. Representative images of H&E-staining in lung tissues of sham air (left) (n=4) and ENDS-exposed (right) (n=4) mice. **(C)** Quantitative morphometric analysis of the alveolar septum of the lung. **(D)** Representative images of Periodic Acid-Schiff staining in lung sections of βENaC mice with or without ENDS exposure. All data were presented at mean ± SEM (student t-test, ** *p*< 0.01).

At 12 weeks of age, mice (n=10 per group) were randomized into either the ENDS exposure group or the control group (sham air). Mice in the ENDS group were exposed to e-cigarette vapor for 1 hour twice daily at a frequency of 5 times per week for a total duration of 10 days as shown in **Figure 1A**. Mice were placed in a smoking chamber attached to an inExpose Smoking Robot (SCIREQ, Montreal, QC, Canada) with an attached e-cigarette accessory (ECX JoyeTech E-Vic Mini, SCIREQ, Canada) for the administration of vaporized S brand e-cigarette liquid containing nicotine salt at 50 mg/ml or sham air (control). During vapor administration, an optimal vapor/air ratio of 1:6 was obtained based on our previous publication (19). After 10 days of ENDS exposure, animals were euthanized within 24 hours after the last e-cigarette vapor exposure using CO_2_. Bronchoalveolar lavage (BAL) fluid and lung tissues were collected for analysis as described below.

### Bronchoalveolar lavage fluid and cytokine measurements

After the mice were euthanized, a tracheostomy was performed, and the lungs were immediately flushed with 1 mL ice-cold phosphate-buffered saline (PBS) twice for collection of BAL fluid. Blood was collected via the abdominal aorta, allowed to sit for 30 min to promote coagulation, and centrifuged at 5,000 rpm for 10 min for isolation of serum. Total cells from the BAL were centrifuged and counted using Countess™ II Automated Cell Counter (Invitrogen, Carlsbad, CA) as previously described (16). Differential immune cell count (5,000 cells/slide) was performed on Shandon cytospin slides (Thermo Shandon, Pittsburgh, PA) stained with Diff-Quik (Dade Bering, Newark, DE). The BAL supernatant and serum samples were stored at -80°C until analysis. Expression of inflammatory cytokines, proteases, and growth factors was assessed in BAL fluid via Mouse XL Cytokine Array (R&D Systems, Minneapolis, MN) following the manufacturer’s instructions to determine the presence of a localized inflammatory response within the lung.

### Morphometric assessment

In order to determine the effects of ENDS exposure on the development of emphysema, we did a morphometric assessment according to the previous protocol (17, 20). Briefly, the mouse lungs were inflated with 1% low melting-point agarose to 25 cm of fixative pressure. After 48 hours of fixation, lungs were paraffin-embedded, sectioned, and stained with hematoxylin and eosin (H&E). Mean linear intercepts were determined and calculated (21).

### Immunohistochemical, immunofluorescent, and histology analysis

The mouse lungs were inflated with 1% low melting-point agarose to 25 cm of fixative pressure and then fixed with 4% neutral buffered formalin for 48 hours. Later, these tissue samples were embedded in paraffin and sectioned into 4 µm sections using a rotary microtome. For immunohistochemical analysis, tissue sections were dewaxed with xylene and rehydrated with graded concentrations of ethanol, followed by antigen retrieval in 10 mM citric acid solution (pH = 6) for 20 min using a pressure cooker. Endogenous peroxidase activity was blocked by 3% hydrogen peroxide. After blocking with 5% Bovine serum albumin (BSA) for 30 min, the slides were incubated with the primary antibodies against the following proteins at 4°C overnight: α smooth muscle actin (α-SMA) 1:500 (Ab5694; Abcam); Von Willebrand Factor (vWF) 1:300 (A008229; Agilent); CD71 (Ab84036, Abcam), proSP-C (AB3786, Sigma), HOPX (11419-AP, Proteintech). After washing, the sections were incubated with the appropriate HRP polymers and developed with 3–3′ diaminobenzidine solution (DAB substrate kit; Vector Laboratories). After counterstaining with hematoxylin, the slides were dehydrated and mounted with a mounting medium. For immunofluorescent staining, after incubated with primary antibodies, the sections were incubated with fluorescent secondary antibody Goat anti-Mouse IgG 488 (A11001, Thermo Scientific), Goat anti-Mouse IgG 594 (A11005, Thermo Scientific), Goat anti-Rabbit IgG 488 (A11008, Thermo Scientific), Goat anti-Rabbit IgG 594 (A11012, Thermo Scientific) for 1h at room temperature. Then the sections were mounted with ProLong Diamond Antifade Mounting with DAPI (P36962, Thermo Scientific).

For Hematoxylin and Eosin (H&E) staining, the paraffin slides were baked at 60°C for 2 hours, then dewaxed with xylene and rehydrated with graded ethanol solutions. Next, the slides were incubated with Mayer’s Hematoxylin for 10 min and washed under tap water for 10 min. Slides were then immersed in Eosin for 30 sec and washed under tap water, after which, the slides were dehydrated and mounted with a mounting medium.

Periodic-acid Schiff (PAS; Sigma Aldrich, Saint Louis, MO) staining was used to evaluate mucus accumulation and immune cell number. Masson’s Trichrome (Sigma, St. Louis, MO) and Picro-Sirius Red (Abcam, Cambridge, MA) staining were used to assess collagen deposition in lung tissue. All stainings were performed following the manufacturer’s instructions. All images were taken at 20x magnification using Keyence Fluorescent Microscope (Keyence, Itasca, IL).

### Cell culture

Human airway epithelial cells (16HBE) were a gift from Dr. Pierre Massion (Vanderbilt University) and maintained in DMEM media. 16HBE cells were grown in 1% penicillin/streptomycin with 10% fetal bovine serum. Human Aortic Endothelial Cells (HAEC) were obtained from Invitrogen (Invitrogen, Carlsbad, CA) and cultured in endothelial cell growth medium supplemented with low serum (2% V/V FBS) (PromoCell; Heidelberg, Germany), and both cells were maintained in a cell culture incubator at 37°C and 5%p CO_2_. 16HBE cells were plated at 30,000 cells/well in 6-well plates. Then cells were treated with ENDS liquid (50 mg/ml) or ENDS liquid without nicotine for 48 hours and harvested for analysis of apoptosis by flow cytometry or western blot. Similarly, HAEC cells were cultured in 6-well plates at a density of 2.0 × 10^5^ cells/well, starved overnight, and treated with 50 mg/ml ENDS liquid with nicotine or ENDS liquid without nicotine for 24 hours or 1 mM H_2_O_2_ for 12 hours (positive control) and then subjected to Annexin V/PI apoptosis analysis by flow cytometry.

### Annexin V/PI apoptosis detection with flow cytometry

The cells were detached with accutase solution and washed with PBS and later binding buffer, followed by resuspension in binding buffer at 10^6^ cells/ml. The cell suspensions were incubated with 5 μL of FITC-conjugated Annexin V (V13242, Thermo) for 15 min at room temperature without exposure to light, followed by washing and resuspension in binding buffer. Propidium Iodide (Sigma Aldrich, Saint Louis, MO) was added followed by incubation for 5 min. The cells were acquired using LSRFortessa flow cytometer (BD Biosciences, Durham, NC), and the data was analyzed by FlowJo software (BD, Durham, NC).

### TUNEL assay for apoptosis detection

Terminal deoxynucleotidyl transferase dUTP nick end labeling (TUNEL) assay was performed using VasoTACS *In Situ* Apoptosis Detection Kit (4826–30-K, Trevigen, Gaithersburg, MD) following the manufacturer’s instructions. In brief, after rehydration, tissue slides were incubated with Proteinase K Solution for 20 min at room temperature, followed by quenching for 5 min. 50 μL of Labeling Reaction Mix were added to the tissues and the slides were incubated at 37°C for 60 min, after which the reaction was terminated by adding the stop solution. The slides were covered with Strep-HRP Solution for another 10 min and developed using TACS Blue Label solution. After counterstaining by Red Counterstain C, slides were dehydrated and mounted with the mounting medium.

### Western blot

Cells were lysed with lysis buffer (RIPA buffer from Thermo Scientific, 89900, 1X Halt protease inhibitor cocktail from Thermo Scientific, 78430, and 1X Halt phosphatase inhibitor cocktail from Thermo Scientific, 78426). Mouse tissues were mixed with lysis buffer (300ul of lysis buffer per 5mg piece of tissue) and homogenated with a homogenizer. Cell debris was pelleted for 10 min at 12,000 x g at 4C. After protein concentration quantification by BCA assay (Thermo Scientific 23225), an equal amount protein of each sample was separated using SDS-PAGE and transferred to a nitrocellulose membrane. Membranes were treated with 5% BSA in PBS-T (PBS with 0.1% Tween 20) blocking buffer for at least 1 h at room temperature, then incubated with primary antibody diluted in blocking buffer overnight at 4C. Anti-ACSL4 antibody (Abcam, ab155282, 1:5000 dilution); anti-GAPDH antibody (CST #2118, 1:1000 dilution); Anti-CD71 (Abcam, Ab84036, 1:1000 dilution), anti-Ferritin antibody (Abcam, Ab75973, 1:1000 dilution). Following three 10 min washes in PBS-T, the membrane was incubated with HRP conjugated secondary antibodies, goat anti-rabbit (Thermo Scientific, 65–6120, 1:5000 dilution), or goat anti-mouse IgG antibody (Thermo Scientific, 62–6520, 1:5000 dilution) in blocking buffer for 1 hour at room temperature. Following three 5 min washes in PBS-T, membranes were incubated with Chemiluminescent Substrate (Thermo Scientific, 34580), and bands were visualized using Autoradiography Film (Denville Scientific, E3012).

### Bodipy staining for peroxidation of polyunsaturated fatty acids

Since the peroxidation of polyunsaturated fatty acids (PUFAA) has emerged as a key driver of oxidative damage to cellular membranes leading to ferroptosis (22), we explored the effects of ENDS exposure on peroxidation of PUFA *in vitro*. 16HBE cells were treated with 50ng/ml of ENDS liquid for 24hs or 1uM of RSL3 (positive control) for 6 hours. After the treatments, cells were washed three times for 10 minutes in PBS, and then fixed in 4% paraformaldehyde for 5 minutes. Next, cells were incubated in 0.1mg/ml BODIPY 581/591 (Invitrogen C10445) in DMSO for 30 minutes in the dark, followed by three times of washes. Cells were then mounted with Prolong gold antifade reagent with 4’,6-diamidino-2-phenylindole (DAPI). Images were taken by Keyence Fluorescent Microscope (Keyence, Itasca, IL).

### Mass-spectrometry-based lipidomics analysis

Lipidomic analysis on serum samples was performed at The Harvard Center for Mass Spectrometry according to our previous publication (23). Briefly, 200 µl of serum samples from mice with either ENDS exposure or normal control was mixed with lung tissue was homogenized with 2 mL of pre-cold methanol (MX0486–1, Sigma), then mixed with 4 mL of chloroform (A452–1, Sigma). The mixtures were combined with 1.2 mL of HPLC grade water (WX0001–1, Sigma), and centrifuged at 3000 rpm for 10 min at 4°C. The bottom layer (chloroform phase) was collected for lipidomics analysis using LC-MS on an Orbitrap Exactive (Thermo Scientific) in line with an Ultimate 3000 LC (Thermo Scientific). Each sample was analyzed in positive and negative modes, in the top 5 automatic data-dependent MS/MS modes. Flow rate was set to 100 μL/min for 5 min with 0% mobile phase B, then, switched to 400 μL/min for 50 min, with a linear gradient of mobile phase B from 20% to 100%. The column was then washed at 500 μL/min for 8 min at 100% mobile phase B followed by re-equilibrated at 0% mobile phase B, 500 μL/min for 7 min. For positive mode runs, buffers for mobile phase A contain 5 mM ammonium formate, 0.1% formic acid, and 5% methanol in water, and, while buffers for mobile phase B are made up of 5 mM ammonium formate, 0.1% formic acid, 5% water, and 35% methanol in Isopropanol. For negative runs, buffers consisted of 0.03% ammonium hydroxide and 5% methanol in water for mobile phase A, and, for mobile phase B, 0.03% ammonium hydroxide, 5% water, and 35% methanol in isopropanol. Each of the spectra for each lipid identified with Lipidsearch software (version 4.1.16, Mitsui Knowledge Industry, University of Tokyo) was manually examined for the presence of the head group characteristic fragment, and for the side chain’s fragment if present. Intensity signals are represented as the area of the parent ion. Integrations and peak quality were curated manually before exporting and analyzing the data in Microsoft Excel. Each ID was based on the fragments found in the literature and compiled in the Lipidsearch database.

### Data analysis

In the case of only two groups, two-tailed *t*-tests were performed to assess the differences. Prior to analysis, we confirmed that the data met the assumptions of normality and homogeneity of variances using appropriate tests (Shapiro-Wilk test for normality and Levene’s test for homogeneity of variances). All analyses were carried out using Prism7.0 software (GraphPad Software Inc., San Diego, CA). Values are represented as mean ± SEM. Levels of significance are designated as *p* < 0.05 unless otherwise indicated.

## Results

### ENDS exposure causes alveolar enlargement and destruction in β ENaC mice

The β ENaC mice have been used as a mouse model for induction of COPD (24–26). The βENaC mice, after exposure to cigarette smoke, display key pulmonary abnormalities of COPD, including inflammation, emphysema, and destruction of alveolar tissue, thus are used to study the molecular pathogenesis of COPD (25). One of the features of emphysema is the enlargement of alveolar spaces associated with the destruction of alveolar walls (27). To determine whether ENDS exposure can induce COPD, the β ENaC mice were given whole-body ENDS exposure as indicated (**Figure 1A**). At the end of the treatment, the mice were sacrificed, and lungs were collected for further pathological analysis. We observed that ENDS-exposed mice displayed impaired lung structure integrity with alveolar enlargement, compared with control mice (**Figure 1B**). We further quantified the air space size and found that the free distance between gas exchange surfaces was significantly increased in the lungs of ENDS-treated mice, indicating that ENDS caused lung emphysema in βENaC mice (**Figures 1B, C**). As mucus accumulation is another feature of COPD, we then assessed mucus content using PAS staining. The results showed that ENDS exposure increased the deposition of mucus in airways compared with the control group (**Figure 1D**). Altogether, our results suggest ENDS exposure causes the pathogenesis of COPD in β ENaC mice.

### ENDS exposure enhances inflammation response in β ENaC mice

**Figure 2:**
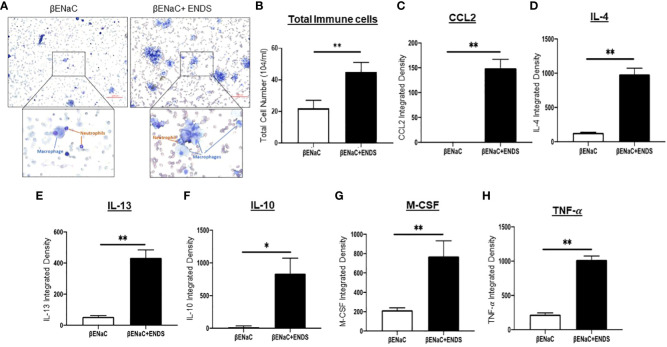
ENDS exposure enhanced inflammation response in β ENaC mice. **(A)** Representative images of Diff-Quik staining of BAL from βENaC mice with or without ENDS exposure (n=3 per group). **(B)** Quantitative analysis of cell number of BAL from βENaC mice with or without ENDS exposure. **(C–H)** Quantification of levels of cytokines/chemokines using Mouse XL Cytokine Array in BAL fluid of βENaC mice with or without ENDS exposure (n=3 per group). *p<0.05; **p<0.01.

COPD is characterized by lung inflammation, which is a critical factor leading to progressive and irreversible airflow obstruction (28). Therefore, we next determined the presence of immune cells in BAL from mice with or without ENDS exposure using the Diff-Quik staining. Clearly, ENDS exposure increased the total number of immune cells including macrophages and neutrophils in BAL (**Figures 2A, B**). We then performed a cytokine array assay to measure cytokine production. Elevated levels of multiple cytokines including CCL2, IL-4, IL-13, IL-10, M-CSF, and TNF- α were found in the lungs of ENDS-exposed mice (**Figures 2C–H**). Interestingly, individual cytokines such as CCL-2, IL-4, IL-10, and IL-13 have been indicated to contribute to macrophage infiltration, M2 macrophage polarization, and pulmonary fibrosis (29–31). Thus, these results indicate ENDS exposure leads to inflammation response in the lung by enhancing immune cell number as well as cytokines productions in the βENaC mice.

### ENDs exposure elevates fibrosis in small airway

**Figure 3:**
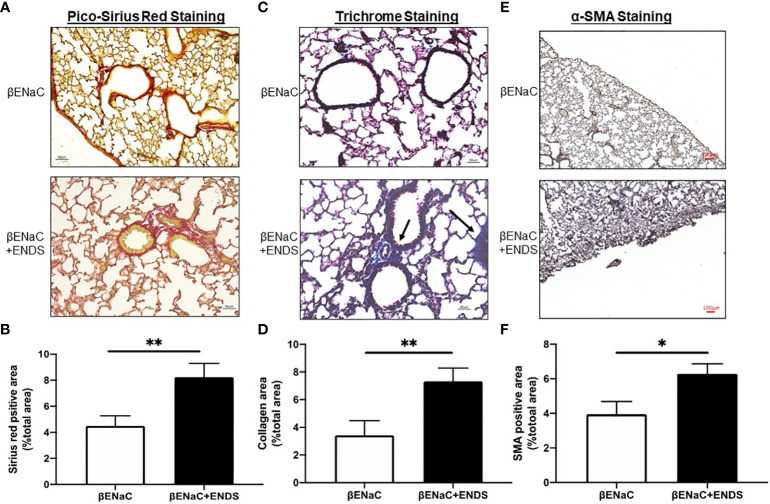
ENDS exposure promoted fibrosis in the small airway. **(A)** Representative images of Pico-Sirius Red staining in lung sections from βENaC mice with or without ENDS exposure (n=3 per group). **(B)** Quantitative analysis of Sirius Red staining as in **(A)** (n=3 per group). **(C)** Representative images of Masson’s Trichrome staining in lung sections from βENaC mice with or without ENDS exposure (n=3 per group). and **(D)** Quantitative analysis of Trichrome staining as in **(C)** (n=3 per group). **(E)** Representative images of α -SMA immunohistochemistry staining in lung sections from βENaC mice with or without ENDS exposure. **(F)** Quantitative analysis of α -SMA staining as in **(E)** by Frida software. The positive staining areas were calculated as percentages of the total area, using five randomly selected sections per mouse, a total of 5 mice in each group. * *p*<0.05; **p<0.01.

Lung fibrosis with excessive deposition of collagen in airways was identified in patients with COPD (32, 33). It has been shown that lung fibrosis promotes airway obstruction thus exacerbating COPD (34). Therefore, we examined whether ENDS affected lung fibrosis during COPD progression. We first assessed the deposition of collagen by Picro-Sirius red staining (**Figure 3A**) and Masson’s Trichrome staining (**Figure 3C**) using lung tissues from mice with or without exposure to ENDS. The results showed that exposure to ENDS elevated total collagen deposition in βENaC mice within the airway and lung parenchyma compared with the control (**Figures 3A–D**). Myofibroblasts, the major player in collagen synthesis, is a well-known hallmark of fibrosis. We therefore examined the existence of myofibroblasts by staining α-SMA, a widely used myofibroblast maker. The results demonstrated that ENDS exposure increased the α-SMA^+^ myofibroblast population (**Figures 3E, F**). Thus, these data indicate that exposure to ENDS enhances collagen deposition and the number of collagen-producing myofibroblasts, revealing that ENDS exposure promotes fibrosis in the lung.

### ENDS exposure induces cell death of endothelial and epithelial cells in the lung

**Figure 4:**
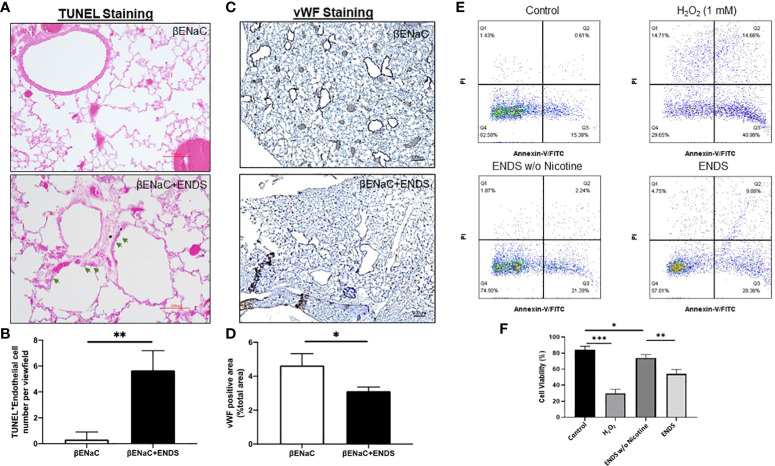
ENDS exposure caused lung vascular injury by inducing endothelial cell death. **(A)** Representative images of H&E and immunochemical staining of TUNEL (TdT-mediated dUTP nick end labeling, blue; counterstained by Red Counterstain C, red) in lung sections from βENaC mice. Green arrowheads indicate TUNEL-positive cells. **(B)** Quantitative analysis of TUNEL staining as in **(A)** (n=3 per group). **(C)** Representative images of vWF (von Willebrand Factor) immunohistochemical staining of lung tissue from βENaC mice with or without ENDS exposure. **(D)** Quantitative analysis of vWF staining as in **(C)** by Frida software. The positive staining areas were calculated as percentages of the total area, using five randomly selected sections per mouse, a total of 5 mice in each group. **(E)** Annexin V/PI apoptosis analysis in human aortic endothelial cells (HAEC). HAEC were treated with ENDS w/o nicotine (bottom left) and ENDS with nicotine (bottom right) for 24h or 1 mM H_2_O_2_ (top right) for 12 hours as positive control and then processed for Annexin V/PI apoptosis analysis by flow cytometry. **(F)** Quantification of Annexin V/PI assay. The cell viabilities were quantified as the Annexin V/PI double-negative cell percentages of total acquired cells. Columns and error bars represent means ± SEM, n=4 *p<0.05; **p<0.01; ***p<0.0001.

**Figure 5:**
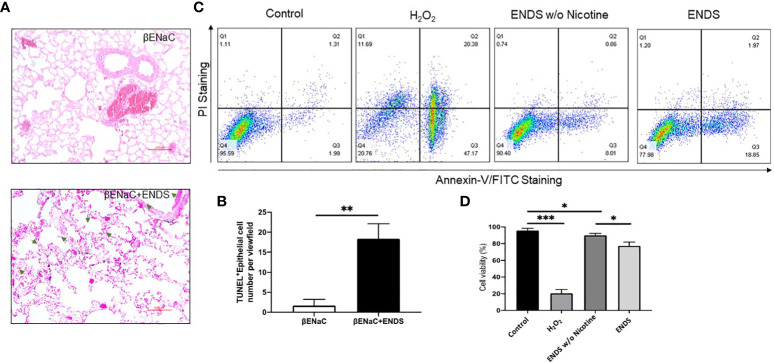
ENDS exposure caused lung epithelial cell apoptosis. **(A)** Representative images of immunochemical staining of TUNEL (TdT-mediated dUTP nick end labeling, blue; counterstained by Red Counterstain C, red) in lung sections from βENaC mice. Green arrowheads indicate TUNEL-positive cells. **(B)** Quantitative analysis of TUNEL staining as in **(A)** (n=3 per group). **(C)** Annexin V/PI apoptosis analysis in human lung epithelial cells (16HBE). The 16HBE cells were treated with, ENDS w/o nicotine and ENDS for 24h or 1 mM H_2_O_2_ for 12 hours as positive control and then processed for Annexin V/PI apoptosis analysis by flow cytometry. **(D)** Quantification of Annexin V/PI assay. The cell viabilities were quantified as the Annexin V/PI double-negative cell percentages of total acquired cells. Columns and error bars represent means ± SEM, n=4 *p<0.05; **p<0.01; ***p<0.0001.

Next, we try to elucidate how ENDS could promote COPD in the lung. Cell death is reported to be a major contributor to the pathogenesis of COPD and there is increased evidence showing enhanced levels of cell death of alveolar epithelial and endothelial cells in the lungs of COPD patients (35, 36). Therefore, we examined total cell death levels in lung tissues using TUNEL staining. The results demonstrated an elevated TUNEL staining of endothelial cells and airway epithelial cells in ENDS-exposed mice compared with control mice, respectively (**Figures 4A, B**, **5A, B**). We observed that the number of endothelial cells, stained by von Willebrand Factor (vWF), a widely used biomarker for vascular endothelial cells, was significantly diminished in ENDS-exposed mice compared with the control mice (**Figures 4C, D**). To confirm this data *in vitro*, we employed the human aortic endothelial cells (HAEC) and tested the effects of ENDS on endothelial cells *in vitro*. As shown in **Figure 4E**, the cell viability was significantly decreased in the nicotine-containing ENDS liquids treated group (57%, **Figure 4E** bottom right) compared with the control group (82%, **Figure 4E** top left). Interestingly, ENDS liquid without nicotine also induced remarkable cell death (75%, **Figure 4E**). On the other hand, ENDS liquid with nicotine and without nicotine significantly led to cell death compared with the control group in airway epithelial cells (16HBE) (**Figures 5C, D**).

### ENDS exposure induces lipid dysregulation and ferroptosis in mice

**Figure 6:**
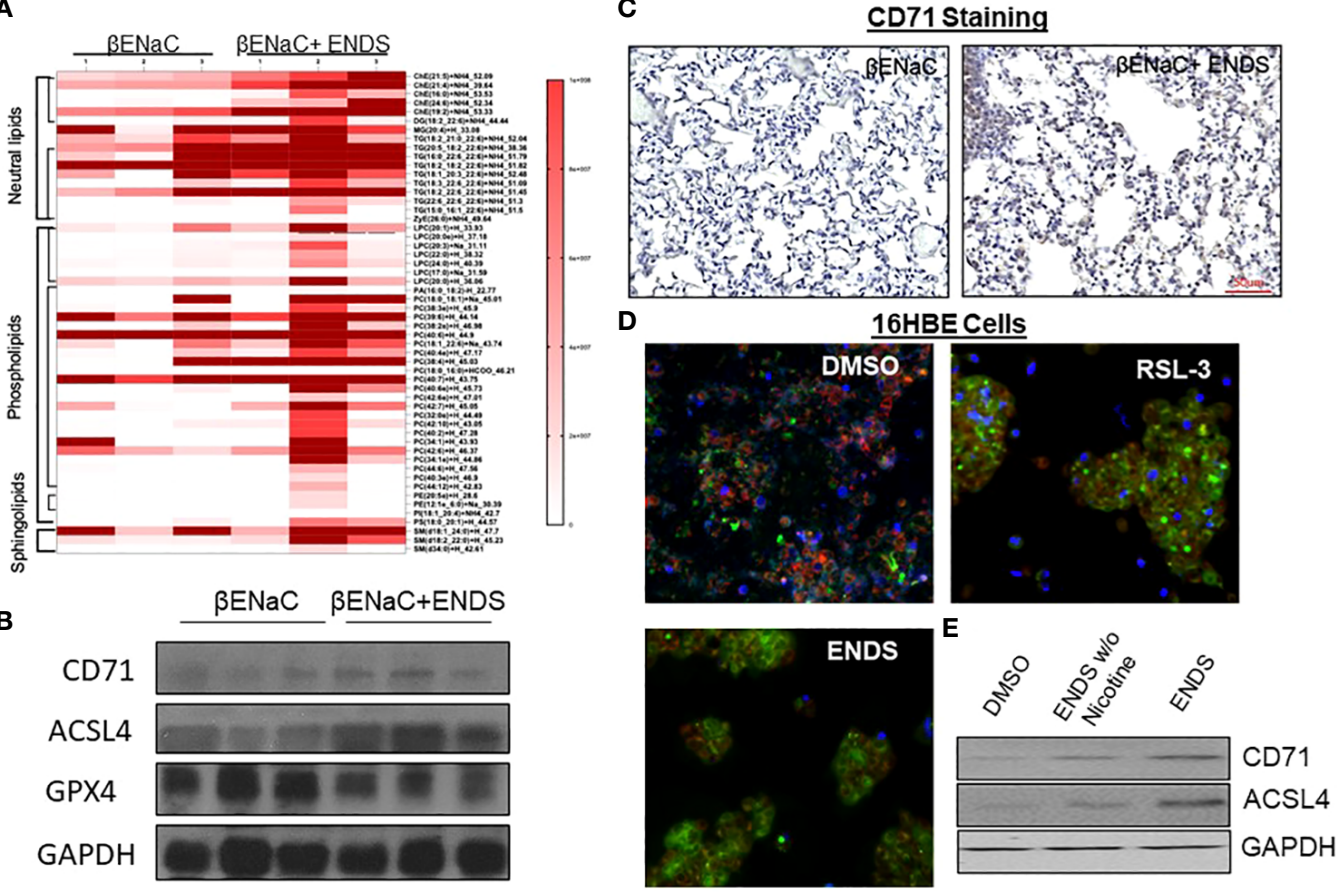
ENDS exposure induced ferroptosis of epithelial cells. **(A)** Mass-spectrometry-based lipidomics analysis using tissues collected from mice with or without ENDS exposure (n=3 per group). **(B)** Western blot determination of expression levels of ferroptosis-specific markers Transferrin Receptor/CD71 and ACSL4 using mouse tissues with or without ENDS exposure (n=3 per group). **(C)** Representative images of CD71 immunohistochemical staining of lung tissues from βENaC mice with or without ENDS exposure. **(D)** BODIPY-C11 assay determining the number of lipid peroxides in cellular membranes in 16HBE cells treated with DMSO (control), RSL-3(Positive control), or EDNS. **(E)** Western blot determination of expression levels of CD71 and ACSL4 in 16HBE cells treated by DMSO or ENDS or ENDS without Nicotine.

**Figure 7:**
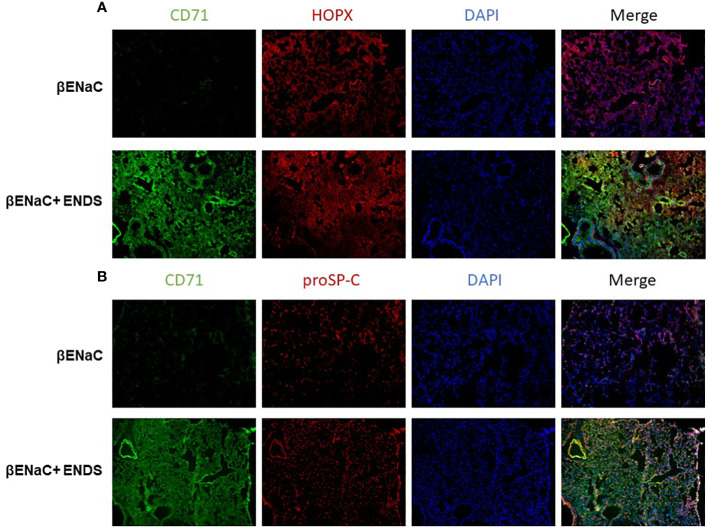
ENDS exposure induced ferroptosis of type I and type II alveolar epithelial cells *in vivo*. **(A)** Representative images of CD71 and HOPX co-immunofluorescent staining of lung tissues from βENaC mice with or without ENDS exposure. **(B)** Representative images of CD71 and proSP-C co-immunofluorescent staining of lung tissues from βENaC mice with or without ENDS exposure.

We noticed that the level of cell death detected by TUNEL staining (either apoptosis or necrosis) does not fully match with lung tissue destruction by ENDS as shown in histological staining. We, therefore, questioned whether there are other formats of cell death mechanisms contributing to tissue mass loss during the progression of ENDS-induced COPD. Besides apoptosis, ferroptosis has drawn intensive attention recently as a critical player in COPD pathogenesis and growing evidence has shown involvement of epithelial cell ferroptosis in COPD pathogenesis (37, 38). Ferroptosis is also reported to induce the release of pro-inflammatory cytokines, leading to COPD-related airway remodeling and emphysema (37), which matches our observation of the elevated level of pro-inflammatory phenotype in ENDS-exposed mice. So we speculated ENDS exposure might induce ferroptosis. We performed a mass-spectrometry-based lipidomics analysis using serum samples collected from mice with or without ENDS exposure. The result demonstrated that relative abundances of more than 50 lipid species were altered after ENDS exposure (**Figure 6A**). We identified increases in the level of 9 species of TAGs and 9 major phospholipid classes (with the total number of detected molecular species of 34 distributed between the following major classes: 22 species; phosphatidic acid (PA); lysophosphatidylcholine (LPA) 7 species; phosphatidylethanolamine (PE), 2 species; phosphatidylinositol (PI) 1 species; phosphatidylserine (PS); 1 species, phosphatidylcholine (PC); 1 species) (**Figure 6A**). Furthermore, we measured the expression of biomarkers of ferroptosis using mouse tissues and found ENDS increased expression of ferroptosis-specific markers including Transferrin Receptor/CD71 (39) and ACSL4 (40) and reduced the expression of GPX4 (**Figure 6B**), the loss of which is reported required for ferroptosis (41). Furthermore, IHC staining of CD71 showed ENDS-treated mice have elevated levels of CD71 in the lungs (**Figure 6C**, **Supplementary Figure 1A**). Then we treated the human bronchial epithelial cell line (16HBE) with or without ENDS (RSL3 as a ferroptosis positive control) and measured ferroptosis by determining the amount of lipid peroxides in cellular membranes using the BODIPY-C11 probe. The data showed that ENDS-treated 16HBE has increased BODIPY-positive ferroptosis cells (**Figure 6D**). The western blot results further supported that ENDS increased the expression level of CD71 and ACSL4, confirming ENDS could induce ferroptosis in bronchial epithelial cells (**Figure 6E**). In order to verify the impact of ENDS on ferroptosis in epithelial cells, we performed immunofluorescent staining with ferroptosis biomarker, CD71. We found ferroptotic type I alveolar epithelial cells (CD71^+^HOPX^+^) and ferroptotic type II alveolar epithelial cells (CD71^+^proSP-C^+^) are both elevated after ENDS exposure (**Figures 7A, B**, **Supplementary Figures 1B, C**), suggesting that ENDS induced ferroptosis in epithelial cells, which is partially responsible for alveolar degradation and destruction.

## Discussion

Cigarette smoking is considered the major risk factor for the development of lung diseases such as COPD and lung cancer in the developed world. Based on the assumption that ENDS vapor contains fewer toxins than conventional cigarette smoke, they were initially considered to be safer (42, 43). While ENDS have a less detrimental effect in terms of environmental pollutants (44) and production of carcinogens (45), the health effects of ENDS usage on chronic lung conditions, such as COPD, and associated public health recommendations remain scant. Recent data classified ENDS aerosol as unsafe (43, 46). By February of 2020, the Centers for Disease Control and Prevention reported approximately 3,000 hospitalizations associated with ENDS or vaping, product use–associated lung injury (EVALI) (47). Given the clinical health issues of ENDS in lung pathobiology, our current study was to investigate the effects of ENDS on COPD progression.

The correlation between ENDS use and COPD occurrence has been reported in human patients (48). However, how ENDS exposure induces COPD has not been fully understood. Our study demonstrates that ENDS exposure promotes COPD in mouse models. ENDS triggered inflammation response in βENaC mouse. ENDS exposure increased immune cell number within BAL fluid and cytokine/chemokine productions. Most notably, CCL2, also known as monocyte chemotactic/chemoattractant protein 1 (MCP1), is a soluble factor in driving monocytic infiltration of tissues during inflammatory processes (49). In a previous study, CCL2-producing macrophages are highly activated in patients with COPD, which is associated with increased monocyte migration (49). Therefore, it is possible that ENDS exposure could cause an increase in monocyte infiltration, which may be worth further investigation. Additionally, significant increases in IL-4, IL-13, IL-10, and well-known anti-inflammatory cytokines, were also observed in response to ENDS exposure. IL-4 and IL-13 are known to coincide with the switching of macrophages toward the M2-like phenotype and IL-10 is the typical cytokine released predominately by M2 macrophage. Multiply research groups have reported an alteration of immunophenotypes of macrophages toward M2 phenotype in COPD subjects, with cytokine production skew toward an M2 profile in blood and BAL samples from patients, suggesting M2 macrophages phenotype is a critical player in COPD disease progression (50–52). It is putative that ENDS might be involved in the regulation of M2 macrophages during COPD. Uncovering the effect of ENDS on macrophage regulation could be of great importance to deepen our current understanding of how ENDS promotes COPD.

IL-10 also appears to function as a regulator of fibrotic processes (53). We premised that ENDS exposure might cause a pro-fibrotic response. We found ENDS exposure caused collagen deposition and a significant increase in the expression of α-SMA within the lung tissues (**Figure 3E**), suggesting activation of fibrotic myofibroblasts. However, we did not uncover the detailed mechanism by which ENDS contributes to lung tissue fibrosis, which is worth further investigation.

While the majority of research focus is on the epithelial lining within the airway, recent evidence suggests endothelial dysfunction plays an essential role in the oxidative stress response to ENDS vaping (54). Here, we validated the effects of ENDS exposure on the cell viability of both endothelial cells and epithelial cells. Cell viability was significantly decreased following treatment with ENDS containing nicotine (**Figures 4F**, **5C**). Interestingly, ENDS liquid without nicotine also had an effect on the induction of apoptosis of endothelial and epithelial cells, indicating other components in ENDS besides nicotine might also play a role in triggering apoptosis of endothelial and epithelial cells. Consistent with our findings, Itsaso et al. demonstrated that the way ENDS exposure in mice induces features of COPD is through a nicotine-dependent manner (10). Another recent study found that chronic ENDS exposure without nicotine adversely affects the vascular endothelial network by promoting oxidative stress and immune cell adhesion (54). One of our future works will focus on elucidating which component(s) besides nicotine in ENDS could lead to apoptosis of endothelial and epithelial cells.

It has been reported that traditional cigarettes smoke can induce ferroptosis which leads to COPD pathogenesis (38, 55, 56). However, it is not clear whether ferroptosis is involved in ENDS-related COPD. Our study is, to the best of our knowledge, is the first study to report the association between electronic cigarette and ferroptosis. Additionally, our discovery of ENDS-inducing ferroptosis in a novel mechanism of how ENDS promotes COPD would provide a new targeting strategy to treat ENDS-related COPD. Our mass-spectrometry-based lipidomics analysis on mouse serum demonstrated that relative abundances of more than 50 lipid species were altered after ENDS exposure (**Figure 6A**). There is substantial evidence supporting a strong correlation between ferroptosis susceptibility and the abundance of PUFA-lipids, including TAGs and phospholipids (57–59). In particular, phosphatidylcholines (PC), the most abundant class of lipids among those that were increased after ENDS exposure, are reported to trigger ferroptosis once oxidized (60, 61). Phosphatidic acid (PA) plays a pivotal role in lipid signaling and membrane dynamics, which are crucial in ferroptosis as PA can influence membrane fluidity and the formation of lipid peroxides (62). Lysophosphatidylcholine (LPC) is known to act as a pro-inflammatory mediator and can modulate cell membrane integrity, thereby contributing to the oxidative stress seen in ferroptosis (63). Phosphatidylethanolamine (PE) is particularly significant in ferroptosis; PE-bound polyunsaturated fatty acids (PUFAs) are substrates for lipid peroxidation, leading to the generation of lipid hydroperoxides that drive ferroptotic cell death (64, 65). We further verified that both type I and type II alveolar epithelial cells are prone to ferroptosis after ENDS exposure using immunofluorescent staining with mouse tissues. The molecular mechanism of ferroptosis on alveoli epithelial cells is still under investigation. There is a high correlation between ferroptosis and inflammatory response reported. We observed increased levels of proinflammatory cytokines which support macrophage infiltration. Therefore, a reasonable speculation is the macrophage could induce ferroptotic cell death of epithelial cells which might further drive COPD.

We did not find all the ferroptotic lipid species as in the previous publication (66, 67), this inconsistency might be due to the sample preparation, limits of mass spectrometry detection, or the specificity to ENDS exposure. However, due to the limitation of the genetic background of ENaC mice, further investigation is needed to elucidate the impact of chronic ENDS exposure with or without nicotine on the endothelium in other genetic background mice. In addition, the vulnerability of gender to COPD due to ENDS exposure also needs to be addressed.

Our study’s findings have significant implications for human health, particularly regarding the use of electronic nicotine delivery systems (ENDS) among individuals with chronic obstructive pulmonary disease (COPD). As ENDS usage rises, understanding its impact on COPD is crucial, and our results suggest that ENDS may worsen COPD symptoms and accelerate disease progression. This challenges the view of ENDS as a safer alternative to traditional tobacco products, especially for those with respiratory conditions. Therefore, our findings support the need for stringent ENDS regulations, including comprehensive testing and standardized guidelines for manufacturing and marketing, alongside public health campaigns to educate users about the potential risks of ENDS, emphasizing that they are not necessarily safe for individuals with compromised lung health.

It is essential to acknowledge the limitations of our study to provide a balanced perspective on our findings. One limitation of our study is the extrapolation of data from mouse models to human COPD progression, as differences in respiratory systems and immune responses between species can affect the applicability of these findings to humans. Further research with human subjects is needed to validate our results and understand their implications for human health. Another limitation is the potential variability in ENDS devices and liquids, with different products having diverse designs, power settings, and compositions, potentially leading to varying levels of exposure to harmful substances. Our study focused on specific ENDS products, which may not represent the full range available to consumers. Future research should examine a broader array of ENDS products for a more comprehensive health impact assessment.

## Conclusions

**Figure 8:**
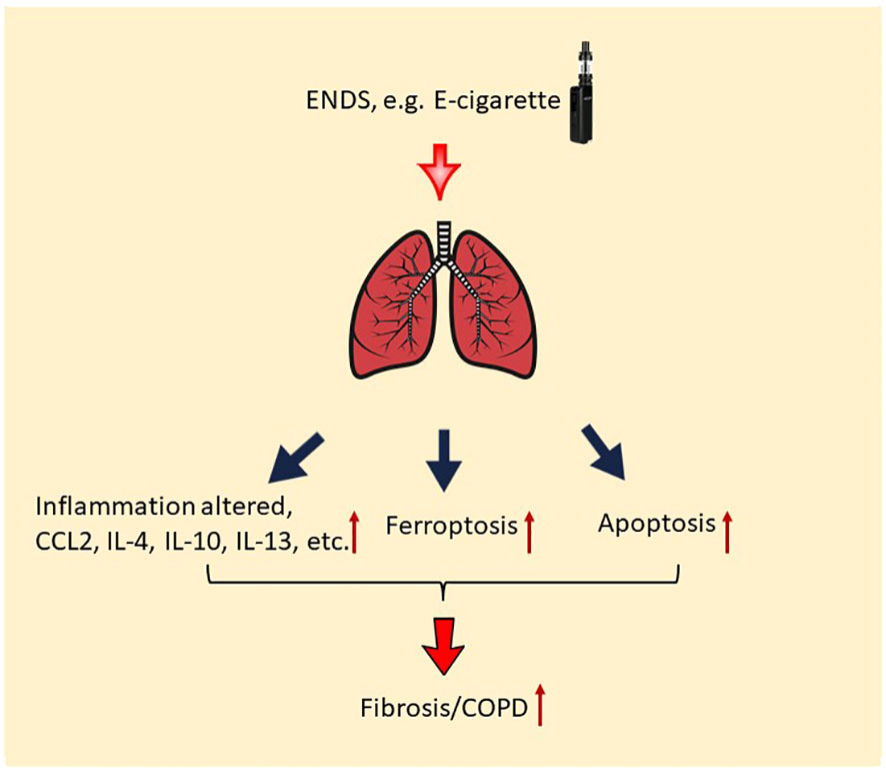
Potential molecular mechanism of the effect of ENDS on COPD. ENDS exposure increases levels of multiple COPD-related cytokines in the lungs, including CCL2, IL-4, IL-10, IL-13, and IL-10; and ENDS promotes fibrosis in the lung. Moreover, ENDS exposure triggers apoptosis of pulmonary endothelial cells and epithelial cells and ferroptosis of epithelial cells. Overall, abnormal lung inflammation, fibrosis, and cell death of endothelial/epithelial cells caused by ENDS suggest that ENDS exposure exacerbates features of COPD.

Taken together, our results demonstrate that nicotine-containing ENDS, in the form of e-cigarette vapor, causes the exacerbation of features of COPD in βENaC-overexpressing mice. Specifically, acute exposure to nicotine-containing ENDS vapor results in mucus accumulation, overproduction of multiple cytokines, deposition of ECM, and fibrosis (**Figure 8**). Last but not least, ENDS exposure induces cell death in epithelium and endothelium in the lung of βENaC-overexpressing mice (**Figure 8**).

## Data availability statement

The raw data supporting the conclusions of this article will be made available by the authors, without undue reservation.

## Ethics statement

The animal study was approved by Georgia State University IACUC Committee. The study was conducted in accordance with the local legislation and institutional requirements.

## Author contributions

HH: Conceptualization, Writing – original draft. MM: Conceptualization, Methodology, Writing – original draft. GP: Data curation, Writing – original draft. YY: Methodology, Writing – original draft. JQ: Formal analysis, Project administration, Writing – review & editing. JY: Conceptualization, Data curation, Funding acquisition, Methodology, Writing – review & editing. Z-RL: Data curation, Supervision, Writing – review & editing. XJ: Data curation, Funding acquisition, Methodology, Writing – original draft, Writing – review & editing.

## Supplementary material

Supplementary Figure 1 Quantification of histopathological findings on ENDS exposure induced ferroptosis **(A)** Quantification of CD71 immunohistochemical staining of lung tissues from βENaC mice with or without ENDS exposure (n=3 per group). **(B)** Quantification of CD71 and HOPX co-immunofluorescent staining of lung tissues from βENaC mice with or without ENDS exposure (n=3 per group). **(C)** Quantification of CD71 and proSP-C co-immunofluorescent staining of lung tissues from βENaC mice with or without ENDS exposure (n=3 per group). Columns and error bars represent means ± SEM, **p*<0.05; ***p<*0.01.
