# Supplementary figures and images for: Inhalation of nicotine-containing electronic cigarette vapor exacerbates the features of COPD by inducing ferroptosis in βENaC-overexpressing mice
Source: Front Immunol. 2024 Jun 14;15:1429946. doi: 10.3389/fimmu.2024.1429946 (PMC11211252; doi:10.3389/fimmu.2024.1429946)

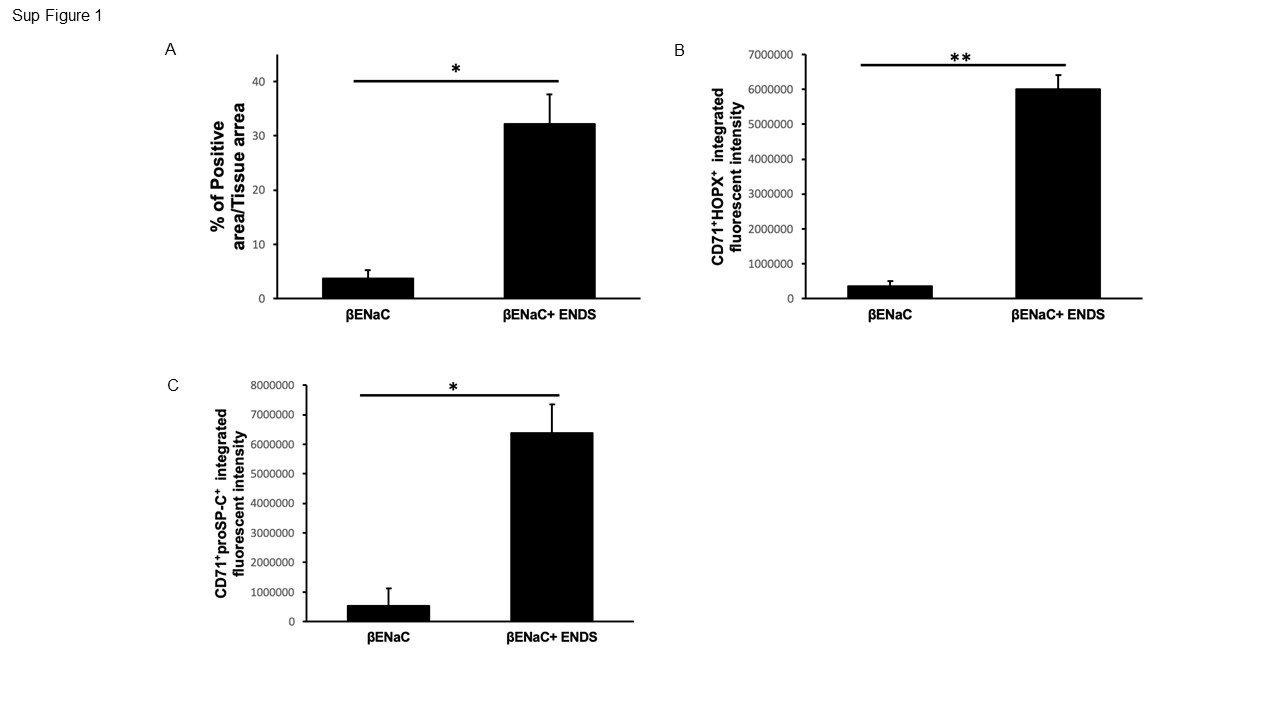

Supplement: Supplementary Figure 1 — Quantification of histopathological findings on ENDS exposure induced ferroptosis (A) Quantification of CD71 immunohistochemical staining of lung tissues from βENaC mice with or without ENDS exposure (n=3 per group). (B) Quantification of CD71 and HOPX co-immunofluorescent staining of lung tissues from βENaC mice with or without ENDS exposure (n=3 per group). (C) Quantification of CD71 and proSP-C co-immunofluorescent staining of lung tissues from βENaC mice with or without ENDS exposure (n=3 per group). Columns and error bars represent means ± SEM, *p<0.05; **p<0.01. [file Image_1.jpeg]
